# Health Education Serious Games Targeting Health Care Providers, Patients, and Public Health Users: Scoping Review

**DOI:** 10.2196/13459

**Published:** 2020-03-05

**Authors:** Nahid Sharifzadeh, Hadi Kharrazi, Elham Nazari, Hamed Tabesh, Maryam Edalati Khodabandeh, Somayeh Heidari, Mahmood Tara

**Affiliations:** 1 Department of Medical Informatics Faculty of Medicine Mashhad University of Medical Sciences Mashhad Iran; 2 Department of Health Policy and Management Johns Hopkins School of Public Health Baltimore, MD United States; 3 Department of Medical Informatics Faculty of Medicine Tehran University of Medical Sciences Tehran Iran

**Keywords:** serious games, health education, health games, game-based learning

## Abstract

**Background:**

Serious educational games have shown effectiveness in improving various health outcomes. Previous reviews of health education games have focused on specific diseases, certain medical subjects, fixed target groups, or limited outcomes of interest. Given the recent surge in health game studies, a scoping review of health education games is needed to provide an updated overview of various aspects of such serious games.

**Objective:**

This study aimed to conduct a scoping review of the design and evaluation of serious educational games for health targeting health care providers, patients, and public (health) users.

**Methods:**

We identified 2313 studies using a unique combination of keywords in the PubMed and ScienceDirect databases. A total of 161 studies were included in this review after removing duplicates (n=55) and excluding studies not meeting our inclusion criteria (1917 based on title and abstract and 180 after reviewing the full text). The results were stratified based on games targeting health care providers, patients, and public users.

**Results:**

Most health education games were developed and evaluated in America (82/161, 50.9%) and Europe (64/161, 39.8%), with a considerable number of studies published after 2012. We discovered 58.4% (94/161) of studies aiming to improve knowledge learning and 41.6% (67/161) to enhance skill development. The studies targeted various categories of end users: health care providers (42/161, 26.1%), patients (38/161, 23.6%), public users (75/161, 46.6%), and a mix of users (6/161, 3.7%). Among games targeting patients, only 13% (6/44) targeted a specific disease, whereas a growing majority targeted lifestyle behaviors, social interactions, cognition, and generic health issues (eg, safety and nutrition). Among 101 studies reporting gameplay specifications, the most common gameplay duration was 30 to 45 min. Of the 61 studies reporting game repetition, only 14% (9/61) of the games allowed the users to play the game with unlimited repetitions. From 32 studies that measured follow-up duration after the game intervention, only 1 study reported a 2-year postintervention follow-up. More than 57.7% (93/161) of the games did not have a multidisciplinary team to design, develop, or assess the game.

**Conclusions:**

Serious games are increasingly used for health education targeting a variety of end users. This study offers an updated scoping review of the studies assessing the value of serious games in improving health education. The results showed a promising trend in diversifying the application of health education games that go beyond a specific medical condition. However, our findings indicate the need for health education game development and adoption in developing countries and the need to focus on multidisciplinary teamwork in designing effective health education games. Furthermore, future health games should expand the duration and repetition of games and increase the length of the follow-up assessments to provide evidence on long-term effectiveness.

## Introduction

### Background

Serious games have emerged as a promising educational technique across various domains [1,2]. Previous studies, including a survey study, have identified health care as one of the main targets of educational serious games [3-5]. In contrast to traditional educational techniques, the focus of serious games on health is partly derived from the fact that they provide individuals with a risk-free environment to practice high-stake tasks and experience unpredictable outcomes. Serious games also provided a unique educational platform to increase patient safety and reduce cost, which, in turn, has propelled the rapid development of new health education games [6,7].

User acceptance is key to the successful impact of educational serious games. Previous studies have assessed various user acceptance challenges of educational games [8,9]. These studies revealed that a wide range of users, including health care providers and medical students, accept serious games as a substantial and useful educational technique [10,11]. These studies also showed that clinical instructors consider serious games as an attractive and engaging educational tool [8,12]. Higher engagement is partly explained by the active learning tasks experienced by the users while interacting with an educational game [13,14].

Similar to other educational techniques, serious games require goal-relevant design, and their effectiveness should be methodologically evaluated [15]. Designing educational serious games requires multiple stages to ensure the engagement of potential end users in all phases of development, ranging from flowcharts and wireframes to multidimensional design and repeated user experience tests [16]. In addition, to increase the generalizability of educational serious games in improving learning objectives, they need to be rigorously evaluated across different user groups using various methods ranging from user studies to focus groups and clinical trials [17].

Several review studies have evaluated the design, development, and outcomes of serious games; however, only a few have focused on health education games [7,15,16]. One study conducted a systematic review of educational serious games for medical students and concluded that serious games should be evaluated before use in medical school curricula [17]. Another study conducted a meta-analysis of sex education serious games and concluded that serious games can be used effectively for promoting sexual health [18]. Moreover, another study evaluated the frequency and progression of health serious games across various domains, including clinical training, rehabilitation, and health education [5]. Considering the current rapid development of serious games, an updated scoping review focusing on health education serious games is lacking.

### Study Objectives

This study offers an updated scoping review of health education games. The study reviews various aspects of the recent developments of educational health games designed for various user groups and provides a comprehensive review of the design characteristics and evaluation of such serious games. The study also addresses the gaps and weaknesses of the recent developments of health education games.

## Methods

### Overall Framework

The York framework was used to develop the general framework of this study [19]. We followed the following stages to guide the methodology of our search and analysis: (1) identifying the research question; (2) identifying relevant studies; (3) search strategy and study selection; (4) extracting information from the studies; and (5) collating, summarizing, and reporting the results.

#### Research Questions

Systematic reviews often focus on specific questions; however, scoping reviews explore questions with a broader scope [20]. In this review, the overarching aims targeted 3 *aspects* of health education serious games: general information, design specifications, and evaluation outcomes. Specific questions targeting each aspect of these health education games were discussed by a group of medical informatics experts (2 faculty members and 4 graduate students) and selected through a consensus process (Table 1).

**Table 1:** Study questions and corresponding health game aspects.

Game aspects	Specific questions
General	What is the frequency of publications per year? How articles are allocated geographically? What are the overall goals of the study?
Design	What are the characteristics of the target groups? What are the types of educational content offered by the games? What types of medical conditions were targeted? What is the distribution of gameplay duration across the studies?
Evaluation	How many repetitions were made in the studies? What was the intervention duration in the studies? What was the duration between the intervention and posttest? What follow-up period was used to evaluate the games? What were the findings of the studies?

#### Identifying Relevant Studies

The research team used an established framework [20] to develop an overall guideline used to identify if a study is considered relevant to the topic of the scoping review. The guide specified additional details about the articles on 3 perspectives of population, concept, and context (Table 2). The research team then applied this guide to develop a detailed inclusion and exclusion criteria for the review process.

**Table 2:** Overall guideline for the inclusion and exclusion of articles.

Items	Description
Population	The search strategy should not limit the educational groups targeted by the studies *(eg, no limitations in terms of gender, age, health, or economic status)*
Concept	The search strategy will not discriminate about the underlying educational goal of the games and will be agnostic about the platform used to deliver the games *(eg, no limitations on the learning techniques and no limits on software choices)*
Context	The search strategy will not limit the inclusion based on the affiliation of authors but will limit the papers to English articles published between January 1985^a^ and December 2018

^a^Previous systematic reviews identified no health education game studies published before 1985.

#### Search and Screening Strategy

The search strategy was guided by the overall inclusion and exclusion framework (Table 2). After reviewing a handful of serious games articles, including review articles, and consulting informatics professionals, the study team developed a set of *potential* keywords to match the overall inclusion and exclusion criteria of the articles (Table 3). To accommodate a wider scope of articles, 3 sets of *potential* keywords were developed to cover gaming, health, and education domains and later were assessed for comprehensiveness while being converted into a unified and single keyword phrase.

**Table 3:** Concepts and potential keywords.

Concepts	Keywords^a^
Game	Game OR (Video Game) OR (Serious Game) OR Gamification OR Gaming OR (Computer-Aided Design) OR (Computer Simulation) OR (Computer Graphics)
Health	Health OR Medicine OR Medical
Education	Education OR Teaching OR Learning OR Training OR Problem-Based Learning OR Computer User Training OR Simulation Training

^a^On the basis of determined keywords and concepts, the search team developed and used the following composite keyword to search for the studies of interest: ((video game) OR (videogame*) OR (serious gam*) OR (gaming) OR (gamification)) AND ((educate*) OR (train*) OR (teach*) OR (learn*)). The publication year of articles was limited from 1985 to 2018, and non-English articles were excluded. Only original studies published in a peer-reviewed venue were included. PubMed and ScienceDirect databases were used to perform the search.

#### Study Selection and Inclusion

**Figure 1:**
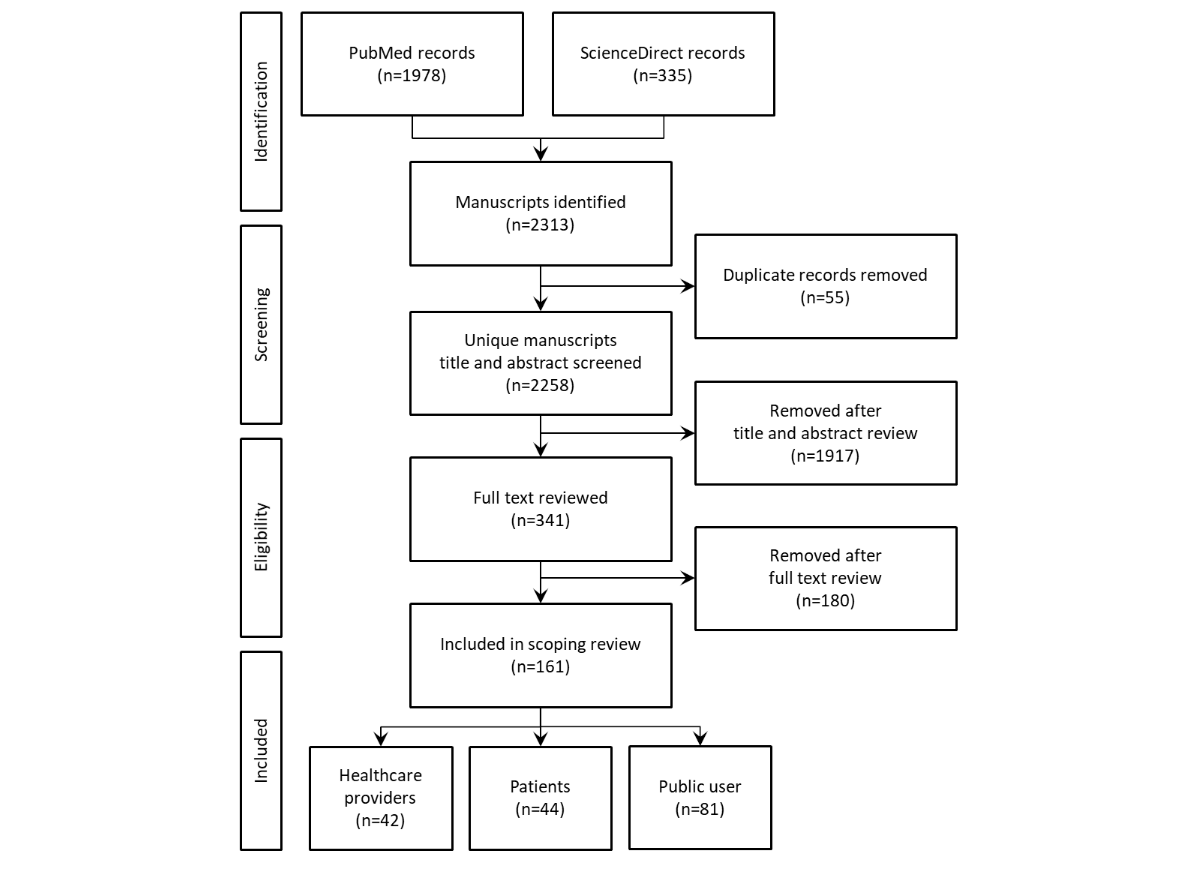
The Preferred Reporting Items for Systematic Reviews and Meta-Analyses diagram of the search methodology.

As depicted in the Preferred Reporting Items for Systematic Reviews and Meta-Analyses diagram in Figure 1 [21], the initial search retrieved 2313 articles from PubMed (n=1978) and ScienceDirect (n=335). After removing duplicates (n=55), 2258 articles were retained for screening. The search team used the inclusion and exclusion guidelines (Table 2) to filter the articles. Screening the title and abstract of the articles resulted in the exclusion of 1917 articles. To harmonize the results and exclude *exercise games*, health games using hardware accessories (eg, sensors) or adopting commercial games that are specifically designed for exercise (with minimal educational content) were removed during the title and abstract screening. The remaining 341 articles were furthered screened after reading their full text, which resulted in 180 articles being excluded from the study. We specifically excluded studies assessing the negative aspects of violent games, as they are not considered health games. The risk of bias was not assessed, as this scoping review did not intend to systematically review and evaluate all effective interventions. In the end, 161 articles were determined to meet all inclusion and exclusion criteria and were fully reviewed in the data extraction phase (which further split the studies based on their target groups of health care providers, patients, and public users).

#### Data Extraction

A data extraction form was developed by the research team after the determination of the conforming variables with the research questions and the study’s goal (Table 4). The variables were categorized based on the study’s 3 major aspects (ie, general information, design, and evaluation; Table 1). The data extraction form was shared with all study team members and was finalized after addressing all remaining questions and comments.

In total, 2 reviewers used the data extraction form to independently mine 9.9% (16/161) of the articles. The kappa coefficient score between the 2 reviewers was calculated at 76%. Both reviewers further discussed discrepancies internally and were trained once more to reach a high consensus rate (ie, reaching a kappa score >90% in the second round of coding). Two reviewers then extracted data from the rest of the articles. In the case of nonconformation between the reviewers, a third reviewer was consulted to reach consensus.

**Table 4:** Data extraction form.

Game aspects	Data element to extract
General	Country Year
Design	Target group Type of study Duration of game Goal of game Goal of study Result of study Specialty of the design team
Evaluation	Intervention evaluation’s tool Game evaluation’s tool Intervention evaluation Game evaluation Follow-up duration Intervention duration The duration between intervention and test

#### Data Collation and Analysis

Data extraction results were collected in 2 Microsoft Excel sheets managed by each of the reviewers. The Excel sheets were then merged to generate the final set of results. Excel functions were used to populate the summary statistics and perform a frequency analysis. To analyze the captured data, we applied a frequency analysis for all variables of interest and presented the results in various chart formats. To improve the interpretability of the results, we stratified all findings into the 3 user groups of health care providers, patients, and public users.

## Results

### Overall Findings

The findings of this review were categorized based on health education serious game aspects and specific questions identified earlier in the review (Table 1). Significant study findings were grouped into the geographic distribution of the studies, publication year, type and goal of the studies, target groups, gameplay duration and repetitions, intervention specs, length of the follow-up period, and the use of multidisciplinary teams.

#### Publication by Geography

**Figure 2:**
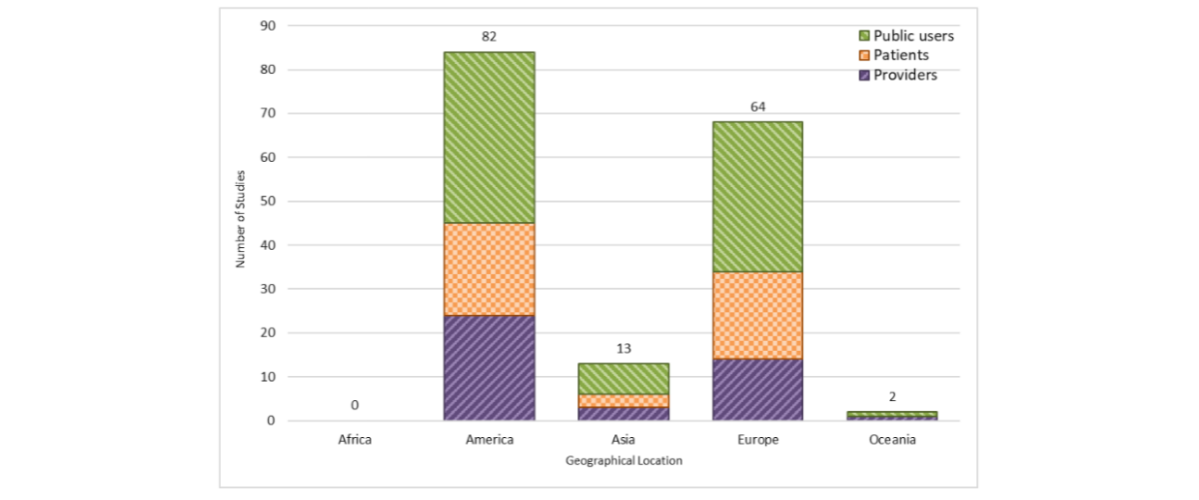
Studies based on geographical locations and stratified by user groups.

Figure 2 depicts the geographical distribution (categorized by continent) of published health education serious game articles. Most of the published articles (n=82) originated from institutions in the American continent (≥90% in North America). After America, Europe (n=64), Asia (n=13), and Oceania (n=2) had the highest number of studies assessing health education games. None of the articles were published by an author affiliated with an institution in Africa.

#### Publication by Year

**Figure 3:**
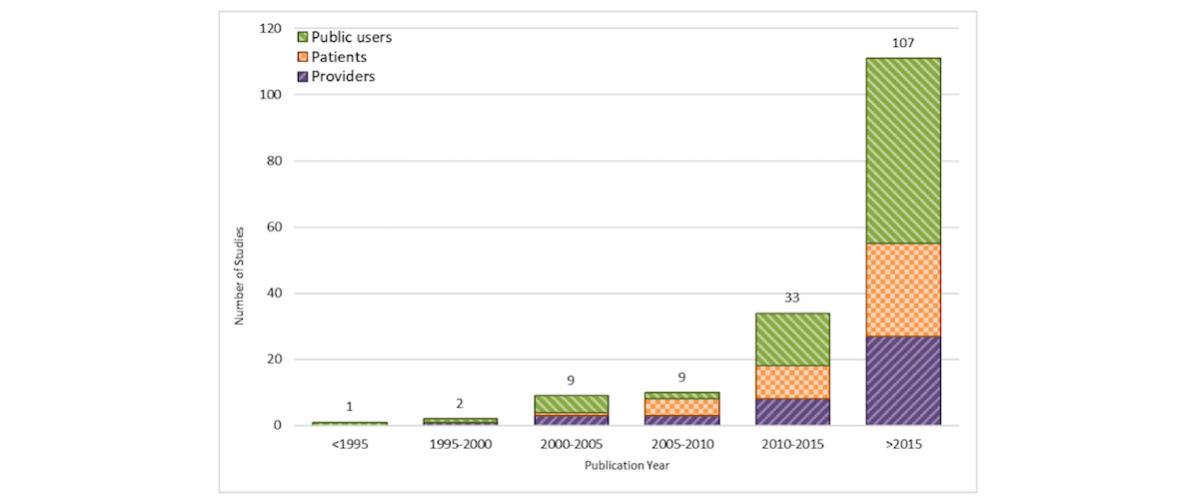
Studies by year and stratified by user groups.

Figure 3 demonstrated the number of articles on health education games per 5-year intervals. The first article was published in 1989. Since 2011, the number of articles has grown considerably, with a notable peak in articles published after 2015 (n=107).

#### Type of Study

**Figure 4:**
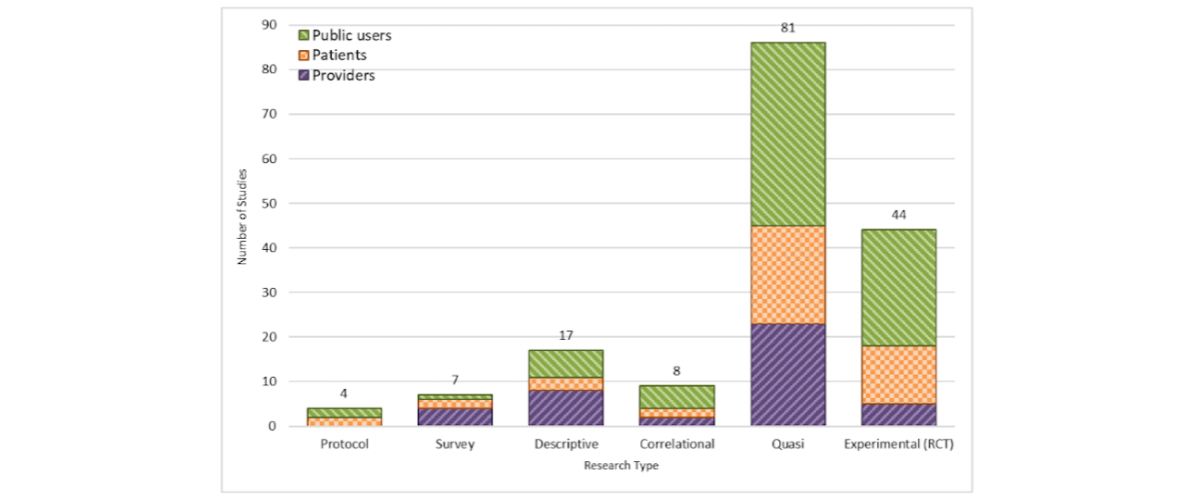
Publications by study design and stratified by user groups. RCT: randomized controlled trial.

In this review, articles were categorized into 2 classes of interventional (77.6%) and observational (22.4%) studies. Among the interventional studies, 44 were randomized clinical trials, whereas 81 of the studies were quasi-experimental (Figure 4). A few studies have assessed the educational health games using a qualitative approach (eg, design protocols and surveys).

#### Study Outcomes

**Figure 5:**
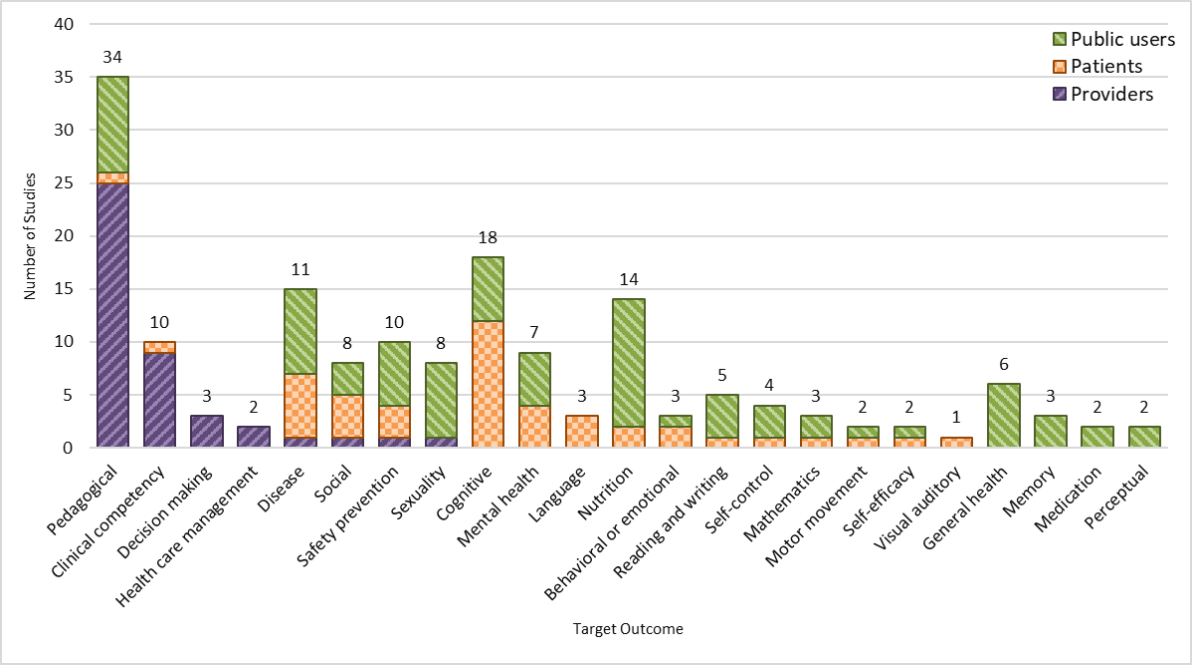
Studies by target outcome and stratified by user groups.

Studies were categorized into 2 classes of knowledge improvement (94/161, 58.4%) and skill improvement (67/161, 41.6%). The knowledge improvement category included multiple subcategories, such as knowledge of diseases (6.8%), general health (6/161, 3.7%), health care management (1.9%), medications (1.2%), mental health (4.3%), nutrition (8.7%), pedagogical content (eg, higher education curriculum; 21.1%), safety and prevention (6.2%), and sexuality (5.0%; Figure 5). The skill improvement category also included multiple subcategories of skills: behavioral and emotional (1.9%), clinical competency (6.2%), cognition (11.2%), decision making (1.9%), language (1.9%), mathematics (1.9%), memory (1.9%), motor movement (1.2%), perceptual (1.2%), reading writing (3.1%), self-control (2.5%), self-efficacy (1.2%), social (5.0%), and visual-auditory (0.6%). Games designed for health care providers were mainly targeting pedagogical knowledge, clinical competencies, and decision-making skills. Games designed for the patients mostly focused on cognition, specific diseases, mental health, social challenges, and a growing number of specific skill sets (eg, self-control and language). Games targeting public users covered a variety of topics, such as nutrition, safety and prevention, and general health items (Figure 5).

#### Target User Groups

We studied 3 specifications of the target groups: age groups, user groups (eg, health care provider, patients, and public users), and medical condition (specific to patients).

#### Age Groups

We used educational age ranges [22] to analyze age groups (Table 5). Some studies only included one of the age categories (78/161, 48.4%), whereas some included more than 1 age category (39/161, 24.2%). Approximately 27.3% (44/161) of studies did not specify the age groups. The studies that were limited to 1 age category included children (36/78, 46%), adolescents (10/78, 12%), adults (29/78, 37%), and elders (3/78, 3%) but did not include neonates or infants or toddlers target groups. Among studies that targeted more than 1 age category (39/161, 24.2%) of the articles, age categories included neonates and infants and toddlers (1/39, 2%), toddlers and children (1/39, 2%), children and adolescents (17/39, 43%), children and adolescents and adults (3/39, 7%), children and adults (1/39, 2%), adolescents and adults (5/39, 12%), adolescents and adults and elders (1/39, 2%), and adults and elders (10/39, 25%). There were no studies to evaluate neonate, infant, and toddler target groups independently.

**Table 5:** Age-specific considerations in patient care and health education.

Groups	Age ranges
Neonates	1 day to 28 days
Infants	29 days to 2 years
Toddlers	1 years to 3 years
Children	3 years to 12 years
Adolescents	13 years to 18 years
Adults	19 years to 65 years
Elders	≥65 years

**Figure 6:**
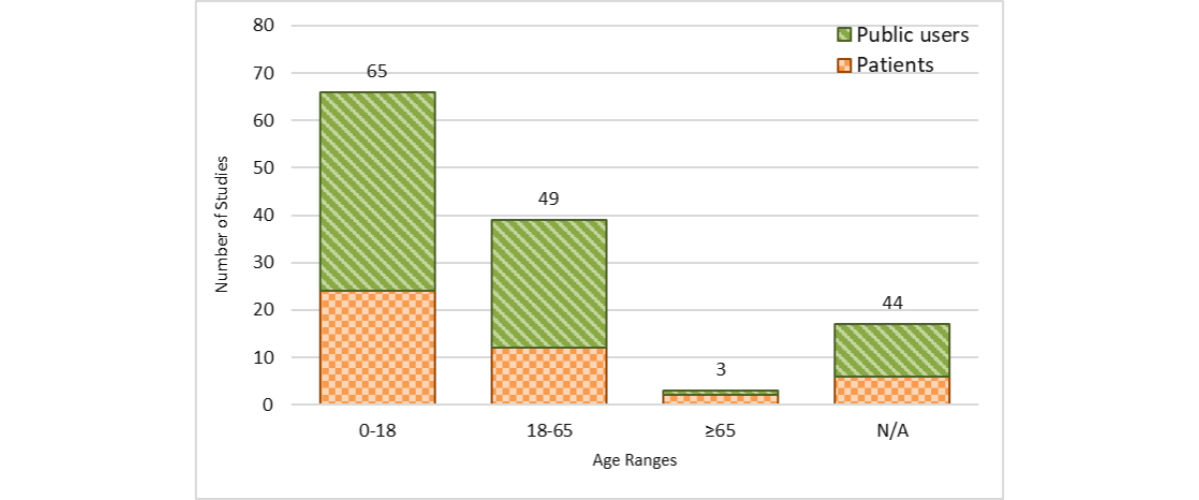
Studies by age ranges of health games designed for patients and public users. Educational health games designed for healthcare providers are not shown (n=42). N/A: not applicable.

After combining the age groups into larger age bins for games targeting patients and public users, the age bin of 0 to 18 years showed the highest number of studies (n=65), compared with 18 to 65 years (n=49) and 65 years and older (n=3, Figure 6). Logically, all games designed for health care providers fell within the age range of 18 to 65 years (not shown in the figure).

#### User Groups

Overall, the user groups were divided into 3 general categories (Figure 1): (1) health care providers, such as physicians and nurses (42/161, 26.1%), (2) patients (38/161, 23.6%), and (3) public users (75/161, 46.6%). A total of 6 studies (6/161, 3.7%) included both patients and public users.

#### Medical Conditions

Of all reviewed studies, 42 (42/161, 26.1%) studies targeted various medical conditions (Table 6). These conditions were either meant to improve individual health outcomes or prevent specific diseases.

**Table 6:** Targeted medical conditions among different age groups of patients and public users.

Medical conditions	Age group^a^ (years)								
	0-12	0-18	0-40	0-65	13-40	13-65	19-65	19-75	≥65
Addiction	—^b^	—	—	—	1	1	—	—	—
Attention-deficit/hyperactivity disorder	2	—	—	—	—	—	—	—	—
Amblyopia	1	—	—	—	—	—	—	—	—
Aphasia	—	—	—	—	—	—	—	1	—
Autism	2	3	1	—	—	—	—	—	—
Behavioral problems	—	1	—	—	—	—	2	—	—
Blood clots	—	—	—	—	—	—	—	1	—
Cancer	—	—	—	—	—	—	1	—	—
Daytime wetting	—	1	—	—	—	—	—	—	—
Dementia	—	—	—	—	—	—	—	—	1
Diabetes	—	1	—	—	—	—	—	—	—
Disabled	—	—	—	1	—	—	—	—	—
Dyslexia	1	—	—	—	—	—	—	—	—
Extremely low birth weight	1	—	—	—	—	—	—	—	—
Fetal alcohol spectrum disorders	3	—	—	—	—	—	—	—	—
HIV	1	—	—	—	—	—	—	—	—
Language disorders	—	1	—	—	—	—	—	—	—
Mathematical skills	1	1	—	—	—	—	—	—	—
Neurodevelopmental disorders	—	1	—	—	—	—	—	—	—
Overweight	—	—	—	—	—	—	2	—	—
Pain after surgery	—	—	—	—	—	—	1	—	—
Psychological disorders	1	1	—	—	—	—	—	—	—
Schizophrenia	—	—	—	—	—	1	—	—	—
Specific language impairment	1	—	—	—	—	—	—	—	—
Stroke	—	—	—	—	—	—	2	—	1
Tinnitus	—	—	—	—	—	—	—	1	—

^a^Age groups cannot be combined, as studies did not report enough details.

^b^Not applicable

#### Duration of Gameplay

**Figure 7:**
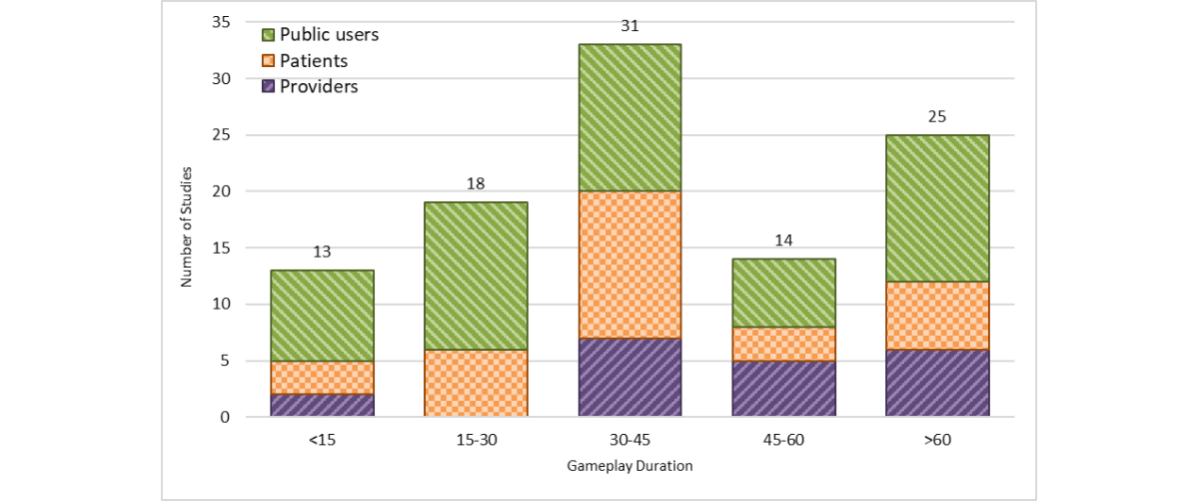
Duration of gameplay in minutes and stratified by user groups. Studies with missing duration of gameplay are not shown (n=60).

Duration of gameplay (ie, time spent on interacting with the game) has been reported in 101 studies (101/161, 62.7%). Figure 7 depicts the distribution of gameplay duration among these 101 studies. The most common gameplay duration was 30 to 45 min (n=31) and the least was less than 15 min (n=13).

#### Number of Game Repetitions

**Figure 8:**
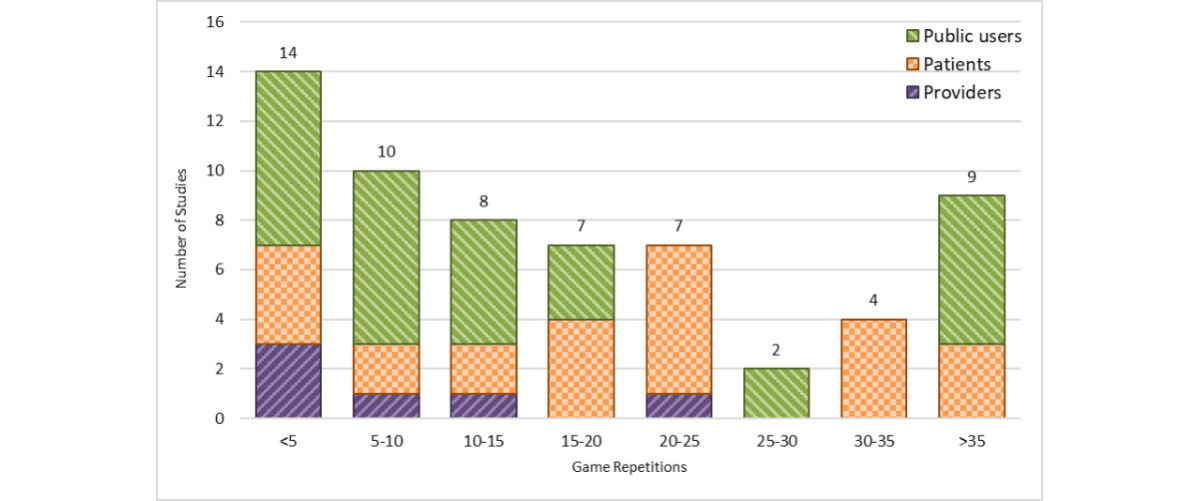
Number of game repetitions across the studies (stratified by user groups). Studies with missing game repetitions are not shown (n=100).

Of all 161 studies, 61 health games (37.9%) mentioned the number of times the game can be repeatedly played (Figure 8). Among these studies, 2 general categories of repetitiveness were observed: (1) articles that limited the number of times a game can be played (n=58) and (2) articles that set no limitations for the number of repeats and users were allowed to have an interaction with the game with unlimited repetitions during the intervention time (n=3; a subset of >35 bar in Figure 8).

Among the articles of the first category, games with less than 5 repetitions had the highest number of articles among health care provider and public user groups. Games designed for patients had 20 to 25 as the highest number of repetitions (Figure 8).

#### Duration of Intervention

**Figure 9:**
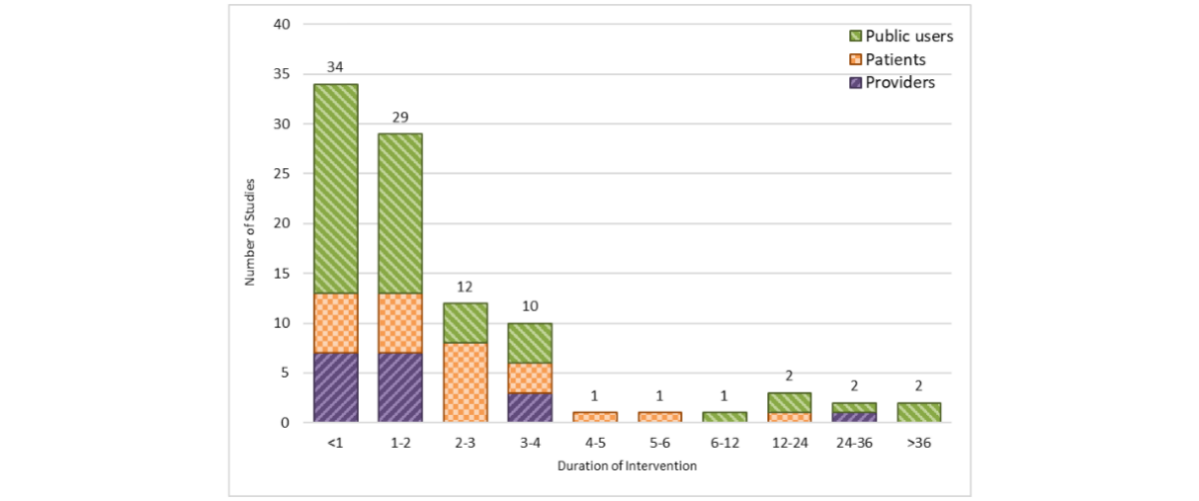
Duration of intervention in months (stratified by user groups). Studies with missing duration of intervention are not shown (n=67).

The duration of intervention was mentioned in 94 of the reviewed studies (Figure 9). The duration of intervention varied between 1 week and 8 years. Most studies (34/94, 36%) had a time range of less than 1 month.

#### Time Between Intervention and Posttest

**Figure 10:**
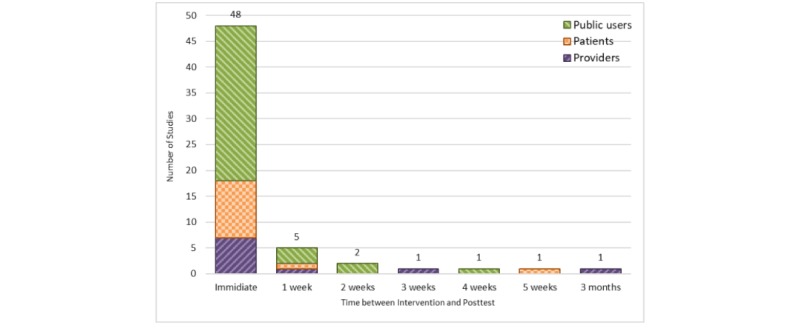
Time between intervention and posttest (stratified by user groups). Studies with missing information are not shown (n=102).

The time between intervention and posttest was reported in 59 (59/161, 36%) of the reviewed studies (Figure 10). Two categories of time between intervention and posttest were seen: (1) conducting a test right after the intervention (48/59, 81%) and (2) conducting a test after at least 1 day is passed from the intervention (11/59, 18%). The time ranged between 1 and 12 weeks among the latter group of studies.

#### Follow-Up Duration

**Figure 11:**
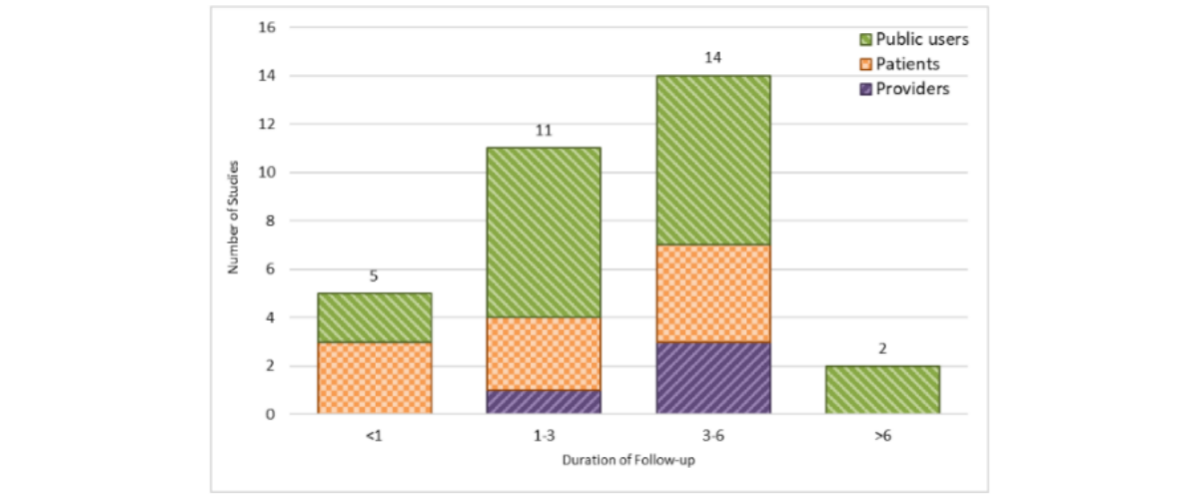
Duration of follow-up in months (stratified by user groups). Studies with missing follow-up duration are not shown (n=129).

Follow-up duration was collected by a few of the studies (32/161, 19%; Figure 11). Among these studies, 97% had a follow-up duration of 1 week to 6 months. Only 1 study reported a 2-year follow-up period.

#### Multidisciplinary Teams

**Figure 12:**
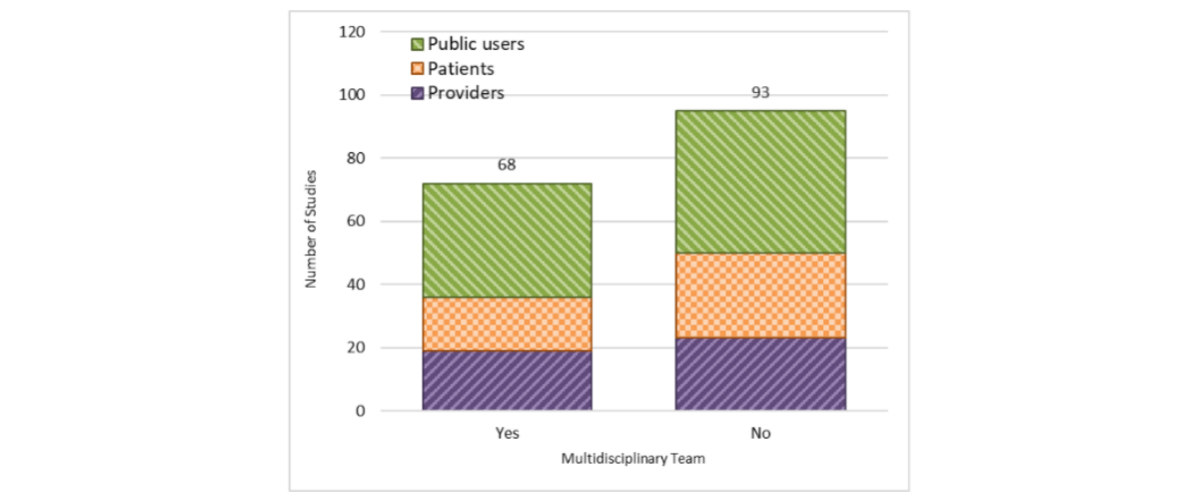
Development of studies by multidisciplinary teams (stratified by user groups).

Although the use of multidisciplinary teams to design health games is strongly recommended, only 42% (68/161) of the reviewed games either explicitly mentioned the use of such teams or implicitly mentioned the involvement of such experts (eg, instructional, clinical, and user experience) in the development and assessment of the games (Figure 12). Analyzing the use of multidisciplinary teams over the years of publications did not show any significant trends.

## Discussion

### Principal Findings

Serious games are increasingly recommended as effective techniques to improve health education [23-25]. Multiple studies have assessed educational serious games in different fields of health, ranging from preventative screening to management of chronic diseases [9,26-28]. Over the last decade, a growing number of these studies have measured the efficiency and effectiveness of serious health education games using randomized trials of patients and clinicians [29-35]. Our review provides an updated scoping review of the underlying patterns and gaps of studies assessing the value of serious games in improving health education for health care providers, patients, and public users.

#### Serious Game Development, Target Groups, and Topics

Our results confirm the concentration of educational health game development in developed countries of North America, Europe, and Asia, thus lacking the opportunity to target educational needs of low-income countries by adopting contextualized or localized serious games. Given the increased availability of smart electronic devices and the penetration of the internet in developing countries [36], more research is needed to develop and assess the impact of serious games targeting specific health educational needs of such populations (eg, infectious diseases) [37]. Given the peak of health game development in recent years (between 2015 and 2018) and the potential higher commercialization of such solutions, focusing on emerging topics of developing countries can benefit larger populations in need [38].

Our results show that more than one-fourth (26.1%) of the health education games focused on higher education needs and targeted medical students and staff. The fact that most of these studies are using health games as an intervention and a considerable number of them have assessed their effectiveness using experimental designs (including randomized trials) provides a growing opportunity to assess the effectiveness of such serious games. It can be anticipated that medical schools (especially in North America and developed countries of Europe and Asia) will gradually incorporate such interactive solutions (ie, serious games) in their common educational curriculum in the near future. In addition, given the low cost of technology needed to use such games in medical and nursing schools, open source or free versions of such serious games can tremendously help reducing educational disparities in clinical sciences among low-resource countries.

An interesting trend revealed by our results is the gradual move from developing disease-specific serious educational games (eg, educational games for diabetes) to targeting broader public health topics (eg, safety and nutrition). Given the impact of health topics such as safety and nutrition on population-level outcomes, the future of educational health games may entail a larger coverage of the general healthy population rather than patients with specific diseases (ie, helping to bridge pubic and population health outcomes [39,40]). The wider target groups of end users can potentially translate into increased market opportunities for educational health games as well as sustainable commercialization over the long term.

#### Game Design and Learning Outcomes

Educational games primarily aim to increase awareness and knowledge among the players. Nonetheless, the ultimate goal of educational *health* games should include a behavioral change in the end users, thus producing a lasting effect. Developing such complex health education serious games, however, is challenging as it requires the participation of multidisciplinary team members to address various game play perspectives, ranging from principles of design to psychology of behavioral change. In this review, the evaluation of the studies revealed that the use of multidisciplinary teams to design health education games is strongly recommended, but it is accomplished occasionally by game developers. Indeed, according to our evaluation, almost half of the studies were deprived from a multidisciplinary team. Continuous collaborations among the members of the multidisciplinary expert team in addressing various aspects of game design and development are strongly recommended to improve the educational experience of the users and potentially improve the impact of health education games.

Most studies reviewed in this study had a short duration of game play with minimal repetitions and limited follow-up periods. In addition, 6 studies did not report an effective intervention and could not achieve desired educational outcomes. These limitations can be attributed partly to the lack of expert user experience designers participating in the study teams [41]. Furthermore, although factors, such as time of intervention, time between intervention and posttest, and duration of follow-up, are critical in achieving long-term knowledge gains, generalized evidence on what factors with what frequency and length works for what age-range is still lacking. More work is needed to established common design guidelines on how to engage different user groups with best game specifications for health education for both patients and medical staff groups.

### Comparison With Prior Work

Previous scoping reviews of health games included all types of serious games, ranging from educational to behavioral change and exercise-focused games [4,5]. Several reviews have predominantly focused on chronic diseases [42-44], whereas other reviews have focused on specific subgroups of educational games, such as games targeting clinical staff and students [10,12,14,45] or patients with distinct medical conditions or diseases (eg, diabetes, asthma, or obesity) [46-53]. Owing to either inclusivity (all health games) or exclusivity (specific educational games) of the previous reviews, these reviews do not reveal the overall trends in educational health games across various target users (ie, health care providers, patients, and public health users). This study provides a fresh review on the latest developments and trends in serious games targeting health education, regardless of the target population or observed clinical context.

### Limitations

Our work had multiple limitations. First, we specifically narrowed our search on educational serious games for health. Our review should not be considered a systematic review of all types of health games. Second, our review did not conduct a statistical analysis to measure significant differences among various target groups (eg, measuring significant differences between patients and public health users). Third, we did not perform a meta-analysis, as the studies did not report enough details about their population to perform such analysis. In addition, the outcomes of interest varied greatly, and a meta-analysis was not appropriate for our review. Fourth, we only included English publications, thus unavoidably excluded studies that might be conducted and published in other developed countries not using English as their primary scientific language. Fifth, the variability of reviewed studies limited our review to encode generic specifications, such as publication date, study location, and target groups. More research is needed to tease out the details reported by various subgroups of these studies (eg, some studies reported exact outcomes of interest). Finally, we used PubMed and ScienceDirect as our search engines; however, other search engines might provide additional studies not indexed in the aforementioned databases.

### Future Work

Future work should include a larger *systematic* review, including additional search engines and, perhaps, focusing on the educational games designed for either patients or clinical providers. Additional work on reviewing educational outcomes of interest among these studies and the effectiveness of the health games in achieving them is also needed.

### Conclusions

Serious games are increasingly used for health education. This study offers an updated scoping review of the studies assessing the value of serious games in improving health education. Most educational health games are still developed in high-income countries, but a surge of new games focusing on healthy behaviors (eg, nutrition and safety) has been observed in the last 5 years. Game developers need to use multidisciplinary teams to improve the design of the serious games in keeping the end users engaged for a longer interaction and potentially more effective educational health outcomes. Investment in serious games, as a low-cost educational tool for both patients and medical providers, can potentially help to fill the gap for health education in developing countries.
